# Structure and Electrocatalytic Properties of Sulfur-Containing Multi-Walled Carbon Nanotubes on a Titanium Substrate Modified by a Helium Ion Beam

**DOI:** 10.3390/nano14231948

**Published:** 2024-12-04

**Authors:** Petr M. Korusenko, Egor V. Knyazev, Alexander S. Vinogradov, Ksenia A. Kharisova, Sofya S. Filippova, Ulyana M. Rodionova, Oleg V. Levin, Elena V. Alekseeva

**Affiliations:** 1 Electrochemistry Department, St. Petersburg State University, 7/9 Universitetskaya nab., St. Petersburg 199034, Russiae.v.alekseeva@spbu.ru (E.V.A.); 2 Department of Physics, Omsk State Technical University, 11 Mira prosp., Omsk 644050, Russia; 3 Laboratory of Physics of Nanomaterials for Chemical Current Sources, Omsk Scientific Centre Siberian Brunch of Russian Academy of Science, 15 Karl Marx prosp., Omsk 644013, Russia; 4 Department of Solid State Electronics, V.A. Fock Institute of Physics, St. Petersburg State University, 7/9 Universitetskaya nab., St. Petersburg 199034, Russia

**Keywords:** sulfur-containing multi-walled carbon nanotubes (S-MWCNTs), helium ion irradiation, scanning electron microscopy (SEM), Raman scattering spectroscopy, X-ray photoelectron spectroscopy (XPS), cyclic voltammetry (CV), electrocatalytic properties

## Abstract

In this work, a set of analytical techniques, including scanning electron microscopy (SEM), Raman scattering spectroscopy, X-ray photoelectron spectroscopy (XPS), energy-dispersive X-ray microanalysis (EDX) and cyclic voltammetry (CV), were used to study the impact of high-energy He^+^ ion irradiation on the structural and electrochemical characteristics of sulfur-containing multi-walled carbon nanotubes (S-MWCNTs) placed on a titanium substrate. The results indicate that the ion beam treatment of the S-MWCNT system led to an increase in the level of imperfections on the surface structures of the nanotubes due to the formation of point defects on their outer walls and the appearance of oxygen-containing functional groups, including SO_x_ groups, near these defects. At the same time, a significant increase in the sulfur concentration (by 6.4 times) was observed on the surface of the S-MWCNTs compared to the surface of unirradiated nanotubes. This was due to the redeposition of sulfur atoms near the point defects under the action of the ion beam, followed by the subsequent formation of direct S–C chemical bonds. Electrochemical studies demonstrated that the irradiated S-MWCNTs/Ti system exhibit enhanced catalytic activity, with improved oxygen reduction reaction (ORR) performance and a substantial increase in anodic current during the oxidation reaction of hydrogen peroxide under alkaline conditions, highlighting their potential for advanced electrocatalytic applications.

## 1. Introduction

The oxygen reduction reaction (ORR) is an important chemical process in many energy conversion and storage systems, such as hydrogen–air fuel cells and metal–air batteries [1,2,3,4]. However, traditional electrocatalysts used for the ORR, which are based on noble metals like platinum (Pt), have several disadvantages, including high costs, poor resistance to pollution and limited durability [5]. In light of this, many research groups are working on the development and application of novel materials with low noble metal contents or non-metallic materials, such as carbon-based materials, with good ORR activity [1,2,3,4,5]. Single-walled (SWCNTs) and multi-walled carbon nanotubes (MWCNTs), which have high electrical conductivity, large specific surface areas and significant flexibility, are widely considered to be some of the most promising catalysts or catalyst supports for these applications [6,7,8,9]. At the same time, MWCNTs are considered to be more promising than SWCNTs because of the variety of their shapes and configurations. Additionally, the process of separating MWCNTs from their bundles and subsequently dispersing them has been well understood, making it less labor-intensive.

Doping of MWCNTs is a practical and powerful approach that can be used to improve their functional characteristics [10,11,12]. Among the various heteroatoms that can be embedded in MWCNT walls, sulfur is one of the interesting dopants but is little used compared to nitrogen and boron [13,14]. Embedding a sulfur atom in carbon nanotube walls provides additional unpaired electrons, leading to a high local spin density and, consequently, high electrocatalytic efficiency for the ORR [15,16,17]. All these factors make sulfur-containing CNTs promising candidates for use as catalysts in various chemical processes.

There are different ways of producing sulfur-containing MWCNTs (S-MWCNTs). First of all, there are Chemical Vapor Deposition (CVD) methods for directly obtaining S-MWCNTs. One of these involves the pyrolysis of sulfur-containing thiophene as a precursor for carbon and sulfur atoms with a Co/MgO catalyst under optimal synthesis conditions (the thiophene vapor concentration ranges from 0.76 to 1% at 1000 °C) [18]. Another CVD method involves the use of liquid dimethyl sulfide (C_2_H_6_S) as a precursor [19]. In this case, a thin layer of Co/MgO catalyst is coated on a silicon wafer and inserted into a horizontal quartz tubular reactor. The reactor is then heated to 1000 °C with a continuous flow of argon and hydrogen. H_2_ is allowed to bubble through liquid C_2_H_6_S to initiate the S-MWCNT growth. In addition, dimethyl disulfide (C_2_H_6_S_2_) is also used for the direct production of S-MWCNTs using the CVD method. This C_2_H_6_S_2_ procedure is more promising than using thiophene and dimethyl sulfide, since the former contains a higher concentration of sulfur (two S atoms in the chain). In this case, the preparation process involves the use of Fe/Y-type zeolite nanoparticles as catalysts, which are placed in a quartz tube furnace and heated to 1000 °C [13]. After that, C_2_H_6_S_2_ is fed for 0.5–2 h under an Ar and H_2_ carrier gas atmosphere to grow S-MWCNTs. This approach allows the achievement of a sulfur concentration of about 0.9 at.% on the surface of the carbon nanotubes.

Other methods for doping MWCNTs with sulfur include wet chemistry techniques [14]. One interesting method involves the sulfonation of pre-functionalized MWCNTs in a 1,3-propane sultone solution with toluene, which is refluxed at 110 °C for 24 h [20]. Another way is based on pre-treatment of MWCNTs in a mixture of concentrated H_2_SO_4_/HNO_3_ (70:30 vol/vol) for 12 h, after which MWCNTs are thiolated by refluxing with phosphorus pentasulfide (P_4_S_10_) in an anhydrous toluene suspension for approximately 200 h (7 days) [21]. The next facile approach that can be used to obtain sulfonated MWCNTs is to add pristine MWCNTs to a solution of acetic anhydride and concentrated sulfuric acid (98%), followed by sonication in an ice bath for 30 min. In the study in question, the amount of sulfur attached to the surface of MWCNTs was found to be 0.39 at.% (1.02 wt.%) [22]. To obtain S-MWCNTs, another simple approach can also be used. This involves adding pristine MWCNTs to chlorosulfonic acid and then heating the mixture in a sealed Teflon autoclave at 180 °C for 5–15 h. The prepared S-MWCNTs are then washed with deionized water until a neutral p*H* is reached, then dried for 12 h at 80 °C. This results in S-MWCNTs with a surface sulfur content of 0.52 at.% [23]. Finally, it is worth mentioning an important study [24] which compared various methods of obtaining S-MWCNTs using wet chemistry techniques, including impregnation following in situ polymerization of poly(sodium4-styrenesulphonate) in the presence of (NH_4_)_2_S_2_O_8_ and deionized water, in situ polymerization of acetic anhydride ((CH_3_CO)_2_O) in the presence of sulfuric acid, thermal decomposition of a 10% ammonium sulfate solution ((NH_4_)_2_SO_4_), and treatment with concentrated sulfuric acid at elevated temperatures. In this research, the highest density of acid groups on the surface of MWCNTs and the best sulfonation of nanotubes was obtained by in situ polymerization of poly(sodium 4-styrenesulphonate).

In conclusion, it should be noted that all the aforementioned methods of preparing S-MWCNTs are multi-step processes that are not highly controlled and do not always involve the use of environmentally friendly substances. At the same time, the concentration of sulfur atoms attached to the surface of MWCNTs, as determined by surface-sensitive X-ray photoelectron spectroscopy (XPS), varies only within the range of 0.39 to 0.9 at.%, depending on the sulfonation method. Therefore, the development of new methods for producing S-MWCNTs with a higher sulfur content is a significant scientific task. The solution to this task could lead to the creation of new materials and devices for electrochemical applications such as oxygen and hydrogen peroxide sensors.

In this paper, we propose a promising method for producing S-MWCNTs with a high degree of sulfur doping. This method involves holding the nanotubes in a concentrated solution of elemental sulfur in ethanol and depositing them on a titanium substrate using layer-by-layer aerosol spraying. After that, the S-MWCNT/Ti system is irradiated with a helium ion beam. The material thus obtained was then tested as an electrocatalyst for the oxidation of hydrogen peroxide (H_2_O_2_) at various p*H* values and the reduction of oxygen in an alkaline electrolyte. 

The main tasks of this work were (i) to perform a comparative experimental study of the morphology, structure and electrocatalytic properties of the MWCNT/Ti and S-MWCNT/Ti systems (initial and He^+^ ion-irradiated) and (ii) to investigate the possibility of using the He^+^ ion-irradiated S-MWCNT/Ti system as an electrochemical sensor for hydrogen peroxide and oxygen. To achieve this, a set of complementary analytical techniques was used, including scanning electron microscopy (SEM), Raman spectroscopy, X-ray photoelectron spectroscopy (XPS), energy-dispersive X-ray microanalysis (EDX) and cyclic voltammetry (CV).

## 2. Materials and Methods

### 2.1. Sample Preparation

Commercially available multi-walled carbon nanotubes (MWCNTs—produced by the Boreskov Institute of Catalysis SB RAS, Novosibirsk, Russia) with a specific surface area of 250 m^2^/g and an outer diameter of 4–24 nm [25] were used in this study. Pure titanium foil (according to the American Standard ASTM for Grade 2), 50 μm thick, was also used. 

To obtain MWCNTs/Ti and S-MWCNTs/Ti systems, suspensions of carbon nanotube powder in ethanol were prepared in the first stage, followed by their dispersion using an ultrasonic bath. Detailed parameters of suspension preparation for the MWCNTs/Ti system are presented in our previous works [26,27]. In the case of the S-MWCNTs/Ti system, a saturated solution of sulfur in ethanol was prepared. For this, rhombic sulfur (S_8_) powder was added to ethanol at room temperature while constant stirring was carried out using a magnetic stirrer until an insoluble precipitate formed. The saturated solution of sulfur in ethanol was obtained by filtering from the precipitate. Then, the MWCNTs powder was mixed with a concentrated sulfur solution in ethanol and allowed to age for 48 h to form S-MWCNTs. Finally, the prepared solution was treated with ultrasound to obtain a uniform dispersion of S-MWCNTs in ethanol.

The deposition of nanotubes on a titanium substrate was performed by the layered aerosol spray technique. In this process, a suspension of MWCNTs, either in pure ethanol or in a saturated sulfur solution, was sprayed onto a heated titanium substrate at 70 °C using an air compressor with a power of 10 W and a capacity of 10 L/min. As a result, a layer of CNTs several micrometers thick was formed on the Ti substrate.

The surface of the S-MWCNTs/Ti system was subjected to ion beam treatment with He^+^ ions with an average energy of 20 keV using the “Composite” ion implanter. The ion beam was formed by ionizing helium atoms with a Penning discharge. The average energy of the ion beam was determined according to the estimated thickness of the MWCNTs layer on Ti, which was calculated based on analyzing the projected range of helium ions in the MWCNTs layer using the SRIM-2013 software code [28]. The irradiation duration of 10 min was chosen based on the results of our previous study [26] to ensure a gentle modification of the MWCNTs layer on the substrate, i.e., a low degree of defectiveness of the layer.

### 2.2. Sample Characterixation

Surface topography information was obtained using a Carl Zeiss Merlin scanning electron microscope (Carl Zeiss Microscopy, Jena, Germany) at the St. Petersburg State University Research Park (Interdisciplinary Resource Centre for Nanotechnology, St. Petersburg, Russia). SEM images were captured at 20 kV with magnifications of 30,000 and 100,000 using an in-lens SE secondary electron detector. EDX microanalysis was conducted using an Oxford Instruments INCAx-act set-up, which was integrated with the microscope. Measurements were taken at 3–6 different points on the surface of each sample and then averaged to obtain data for chemical composition analysis.

Raman spectra were recorded using a SENTERRA spectrometer (Bruker, Billerica, MA, USA) at room temperature with a laser wavelength of 532 nm in the St. Petersburg State University Research Park at the Centre for Optical and Laser Research (St. Petersburg, Russia). The laser power was set to 2 mW for all measurements. All spectra were collected in the range of 100–3600 cm^−1^ with a step of 0.5 cm^−1^. Then, all the spectra were processed, including the removal of the photoluminescence background from the baseline using a quadratic polynomial and subsequently normalizing the spectra to the intensity of the *G* band. In order to assess the degree of defectiveness of the surface layer of nanotubes on a titanium substrate, the ratio of the *D* band to the *G* band was determined, in accordance with the study reported in [29].

The survey and C 1*s*, O 1*s* and S 2*p* core-level (CL) photoemission (PE) spectra were measured using an ESCALAB 250 Xi laboratory spectrometer (Thermo Fisher Scientific, UK) with a hemispherical energy analyzer and monochromatic AlK_α_ radiation (*hν* = 1486.6 eV). The pass energy of the analyzer when recording survey and CL PE spectra was 100 and 20 eV, respectively. The calibration of the binding energy scale was performed using a pure gold foil, with measurements taken for the energy position of the Au 4*f_7/2_* PE line and the Fermi level. The CL PE spectra were carefully examined by fitting them with a set of components using Casa XPS 2.3.16 software code [30]. All XPS measurements were performed at the St. Petersburg State University Research Park (Centre for Physical Methods of Surface Investigation, St. Petersburg, Russia).

Electrochemical studies were conducted using a Bio-Logic VMP3 potentiostat (Seyssinet-Pariset, France) at room temperature. Measurements were performed in a three-electrode cell with a titanium substrate coated with MWCNTs or S-MWCNTs (before and after He^+^ irradiation) as the working electrode. An Ag/AgCl reference electrode was used, and a Pt plate (1 cm^2^) acted as the counter electrode. The active area of each electrode was measured using a caliper prior to the electrochemical measurements. This area was then used to normalize the current densities when analyzing electrochemical data for each experiment. 

The CV method was used to investigate the electrocatalytic properties of the materials in the ORR. Measurements were carried out over a potential range of 0.1 to −1.1 V relative to the Ag/AgCl reference electrode at a scan rate of 10 mV/s for 5 cycles. The experiments were performed in an electrolyte solution (0.1 M KOH) under two different gas conditions: argon (Ar) and oxygen (O_2_). Before each measurement, the electrolyte was saturated with the gases for 30 min. The electrochemical activity of H_2_O_2_ electro-oxidation was investigated by the CV method in standard buffer solutions with different p*H* values of 4, 7 and 9. The CV curves were recorded over a potential range of −0.2 to 1.0 V at different scan rates of 10, 50 and 100 mV/s. The measurements were carried out both in the pure electrolyte and with the addition of H_2_O_2_ at various concentrations (10^−2^ and 10^−3^ M).

## 3. Results and Discussion

### 3.1. SEM and EDX

**Figure 1:**
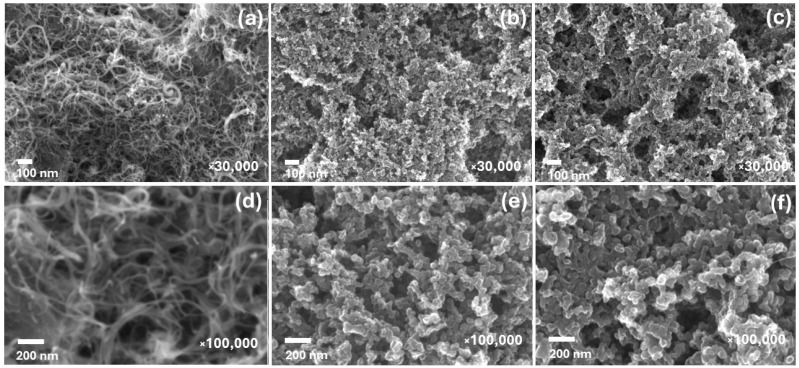
SEM images of MWCNTs/Ti (**a**,**d**) as well as S-MWCNTs/Ti before (**b**,**e**) and after irradiation with He^+^ ions (**c**,**f**) at different magnifications.

Let us first consider the SEM images of the objects under study, which are presented in Figure 1. Upon detailed examination of the SEM images of the MWCNTs/Ti system, it is clearly visible that a continuous layer of nanotubes formed on the surface of the titanium substrate (Figure 1a,d). The measurements performed on 20 individual nanotubes allowed us to conclude that their average outer diameter was approximately 20 nm. In addition, the nanotubes in the layer were arranged predominantly parallel to the substrate and formed many intersections and overlaps. When moving to sulfur-containing MWCNTs on a Ti substrate (Figure 1b,e), there were significant changes in the surface morphology of the sample, apparently related to the process of preparing S-MWCNTs. It is important to note that the S-MWCNTs layer completely covers the titanium substrate and retains a partially porous structure relative to the MWCNTs/Ti system. Additionally, an increase in the average outer diameter from 20 to approximately 30–50 nm was also noted when moving from MWCNTs/Ti to S-MWCNTs/Ti.

Irradiation of the S-MWCNTs layer on the Ti substrate with helium ions resulted in some transformation of the layer surface. Specifically, an increase in the porosity of the surface layer was observed due to the appearance of rounded voids. The formation of these voids was likely due to the partial destruction of nanotubes caused by the high-energy ions (Figure 1c,f). There was also an increase in the outer diameter of individual S-MWCNTs to 60–80 nm relative to the non-irradiated system. This was for two reasons: the welding of nanotubes in some places and the possible sputtering and redeposition of sulfur from one nanotube onto neighboring ones. The welding of the nanotubes was likely due to the creation of structural defects, mainly point defects such as mono- and divacancies, as well as topological pentagon–heptagon (5–7) Stow–Wales defects resulting from the removal of carbon atoms from the nanotube walls under the influence of high-energy helium ions, which leads to an increase in the surface free energy of S-MWCNTs. Previously, in [26,27], we observed a similar welding effect in the MWCNTs/Ti system after treatment with helium ions under similar exposure conditions. 

The elemental analysis data obtained using the EDX method, with an average for each sample calculated over several points, are summarized in Table 1. It can be clearly seen that all samples contain carbon, oxygen and titanium from the substrate. In the case of the S-MWCNTs/Ti system, the presence of sulfur at a level of about 0.5–0.6 at.% was also observed both before and after irradiation. It is important to note that as a result of irradiation of S-MWCNTs/Ti, the sulfur concentration remains practically unchanged, while the concentration of oxygen, on the contrary, increases slightly with a simultaneous decrease in the titanium concentration. In other words, according to EDX data, it can be concluded that irradiation of S-MWCNTs/Ti with a He^+^ beam does not lead to a decrease in the sulfur concentration in the analyzed sample layer. At the same time, the increase in the oxygen concentration in the treated sample can be associated with the fixation of additional oxygen-containing functional groups (OCFGs) in the places of structural defects in the nanotube walls [26].

### 3.2. Raman Scattering Spectroscopy

**Figure 2:**
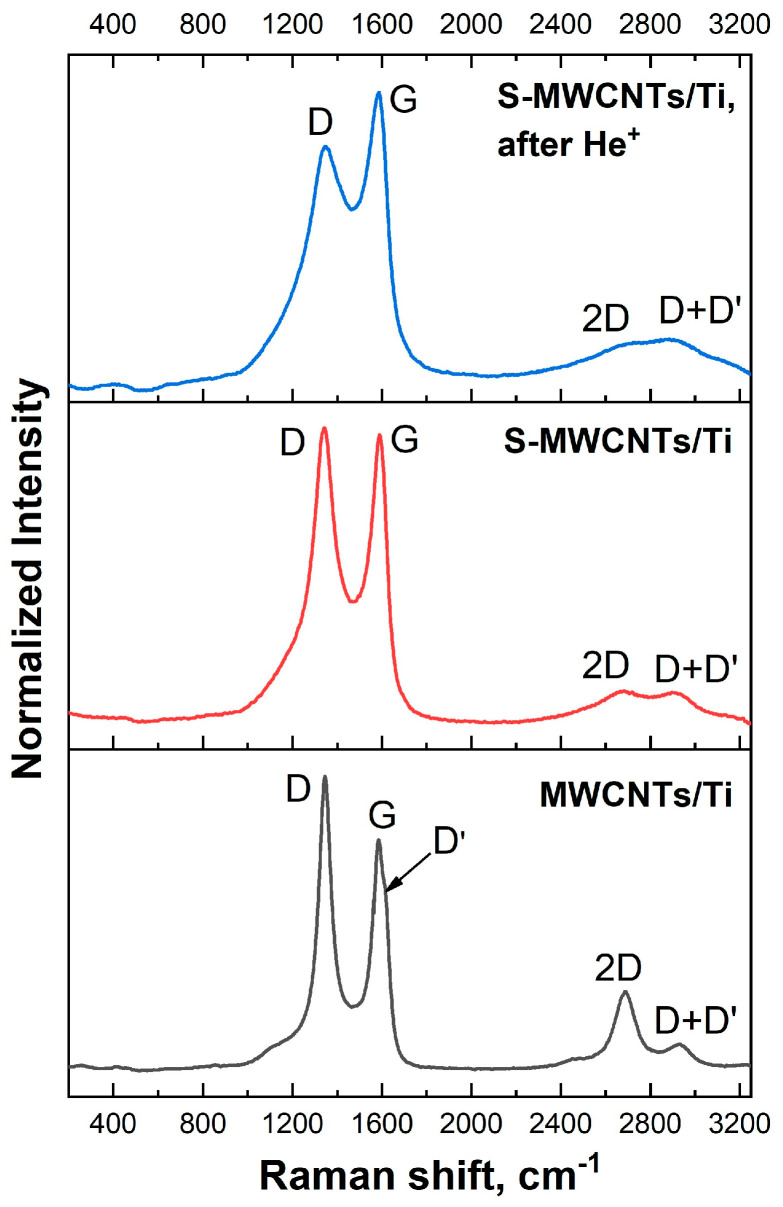
Raman spectra of MWCNTs/Ti as well as S-MWCNTs/Ti before and after irradiation by He^+^ ions.

The Raman spectra of MWCNTs/Ti and S-MWCNTs/Ti before and after He^+^ irradiation are shown in Figure 2. Let us first consider the spectrum of the MWCNTs/Ti sample, which contains the *D*, *G*, *D′*, 2*D* and *D*+*D′* bands. The *G* band at 1579 cm^−1^ corresponds to the E*_2g_* vibrational mode and is observed in all Raman spectra of sp^2^-hybridized carbon atoms [31,32,33]. The *D* band at 1346 cm^−1^ appears only when the symmetry of the carbon lattice is reduced due to the presence of structural defects, as reported in various studies [31,32,33]. Meanwhile, the nature of the *D′* band at ~1605 cm^−1^ still remains unknown, although it appears in all spectra of graphite materials with a low degree of defectiveness, when the average crystallite size is greater than 7 nm, and is absent in those of systems with a smaller crystallite size [29]. In addition, the Raman spectrum of the MWCNTs/Ti sample shows second-order bands: the 2*D* band at 2680 cm^−1^, which is an overtone of the *D*-band, and the *D*+*D′* band at 2930 cm^−1^, which is a combination of the *D*- and *G*-bands [31,34]. 

When moving from the MWCNTs/Ti to the S-MWCNTs/Ti system (the red curve in Figure 2), it can clearly be seen that all bands except for *D′* in the Raman spectrum are present. The main changes are associated with a slight blue shift of the *G* band by approximately 10 cm^−1^, an increase in its intensity and a broadening of the *D* band. In a previous study [31], we found that the blue shift of the *G*-band is due to the addition of OCFGs on the MWCNTs surface after ion beam irradiation. However, in the case of S-MWCNTs/Ti, it is not possible to exclude the formation of SO_x_ groups during sample preparation, which can also affect the position of the *G* band. The broadening of the *D* band is likely due to the presence of new defect states in the nanotubes, the vibrations of which contribute to the spectral region around 1500 cm^−1^. It is most likely that these states are primarily associated with the formation of amorphous carbon and functional group fragments during the preparation of S-MWCNTs [34,35]. Changes in the bands in the second-order region also indicate an increase in the defectiveness of the S-MWCNT wall structure. Specifically, an increase in the intensity and half-width of the *D*+*D′* band can be observed, while the 2*D* band shows a decrease in the intensity and its blurring [34]. It is also important to note that the Raman bands in the region of 200–500 cm^−1^, associated with the vibration of S–S bonds in crystalline sulfur (S_8_), are absent in the spectrum of S-MWCNTs/Ti (Figure 2). This result suggests that sulfur on the surface of MWCNTs is apparently in an amorphous state, which was also observed by the authors of [36].

Irradiation of the S-MWCNTs/Ti system with helium ions results in a significant broadening of the *D* band. This indicates an increase in the degree of imperfection of the nanotube walls due to the creation of new types of defects, such as mono- and divacancies, vacancy clusters, and the presence of OCFGs, including SO_x_ groups near these defects (the blue curve in Figure 2) [26]. An increase in the number of defects in the nanotube walls is also confirmed by a stronger blurring of the 2*D* band and an increase in the intensity of the *D*+*D′* band. At the same time, no shifts of the *D* and *G* bands relative to the untreated S-MWCNT/Ti system were observed.

Finally, let us analyze the change in the peak intensity ratio of the *D* and *G* bands (the so-called I*_D_*/I*_G_* parameter) to assess the degree of defectiveness of the nanotube wall structure in the MWCNTs/Ti and S-MWCNTs/Ti systems before and after irradiation with helium ions. It can clearly be seen (Figure 2, lower panel) that in the Raman spectrum of MWCNTs/Ti the *G* band has a lower intensity compared to the *D* band. This result can be caused by the ultrasonic treatment which produces a homogeneous suspension of the nanotubes in ethanol, which is necessary for the preparation of the MWCNTs/Ti sample. Similar observations have also been made in studies using other solvents [37,38,39]. However, when moving to the S-MWCNTs/Ti samples, the intensity of the *G* band increases and becomes maximal for the sample after ion irradiation. Additionally, it can also be seen that the *D* band is significantly broadened, especially in the case of the spectrum of the S-MWCNTs/Ti after irradiation compared to the similar band in the spectrum of the MWCNTs/Ti sample. In view of this, it is more correct to use the ratio of their integral intensities (band areas) rather than peak band intensities as the I_D_/I_G_ parameter [29]. The values of the I_D_/I_G_ parameters based on integral intensities for MWCNTs/Ti, initial S-MWCNTs/Ti and irradiated S-MWCNTs/Ti are 1.83, 2.57 and 2.44, respectively. In other words, a noticeable increase in the level of defectiveness of the MWCNT walls can be observed when moving from MWCNTs/Ti to S-MWCNTs/Ti samples. At the same time, the slight decrease in the I_D_/I_G_ parameter for the irradiated S-MWCNTs/Ti system may be associated with the transformation of defects and functional groups on the surface of nanotubes under the action of helium ions.

### 3.3. XPS

**Figure 3:**
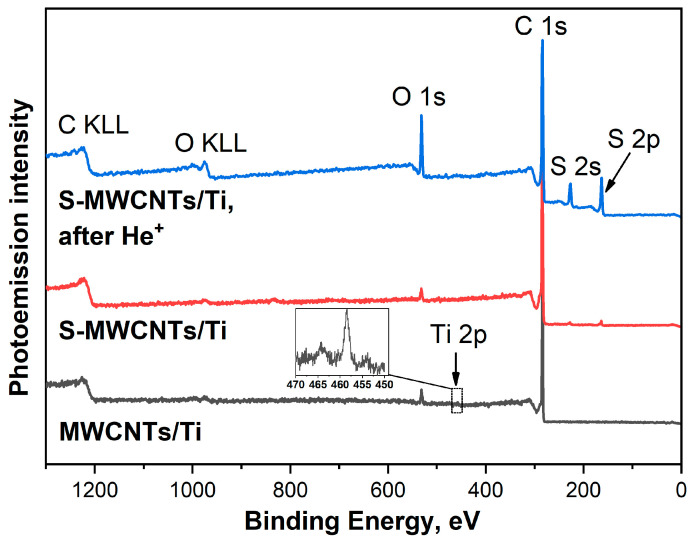
Survey PE spectra of MWCNTs/Ti as well as S-MWCNTs/Ti before and after irradiation by He^+^ ions.

Elemental analysis of the MWCNTs/Ti and S-MWCNTs/Ti systems, both before and after irradiation with He^+^ ions, was performed by the XPS technique by analyzing the intensities of the core PE lines of elements in the survey spectra (Figure 3). The results of this analysis, based on atomic sensitivity factors [40], are summarized in Table 1. In general, the percentage changes for C and O elements observed in the XPS spectra correlate with the changes in the EDX data, whereas these changes for S and Ti elements are different (see Table 1). However, when comparing the outcomes of the XPS and EDX methods, it is important to consider that XPS is a surface-sensitive technique that analyzes a sample up to a depth of 3–5 nm, while EDX examines a layer 1.2–1.5 μm thick. Therefore, the EDX data show a significant presence of Ti atoms from the substrate, resulting in a higher concentration of Ti and lower concentrations of C and O when compared to XPS. Based on the comparison of EDX and XPS data, it follows that the sulfur concentration, as measured by the EDX method, for both the initial and irradiated S-MWCNTs/Ti is almost the same and is approximately 0.5–0.6 at.%. However, the XPS results show that after irradiation, the sulfur concentration in the near-surface layers of the nanotubes increases from 1.68 to 10.79 at.%, i.e., by a factor of 6.4. Based on this, it is reasonable to assume that irradiation with a He^+^ ion beam leads to the sputtering of sulfur, concentrated in the form of agglomerates on the surface of nanotubes, and its redeposition on neighboring nanotubes as a thin layer. The XPS data also show a significant increase in oxygen content after irradiation of the S-MWCNTs/Ti system. This may indicate the formation of point defects in the surface walls of the MWCNTs, an increase in the free energy of the nanotube surfaces and the attachment of OCFGs to them [26]. 

**Figure 4:**
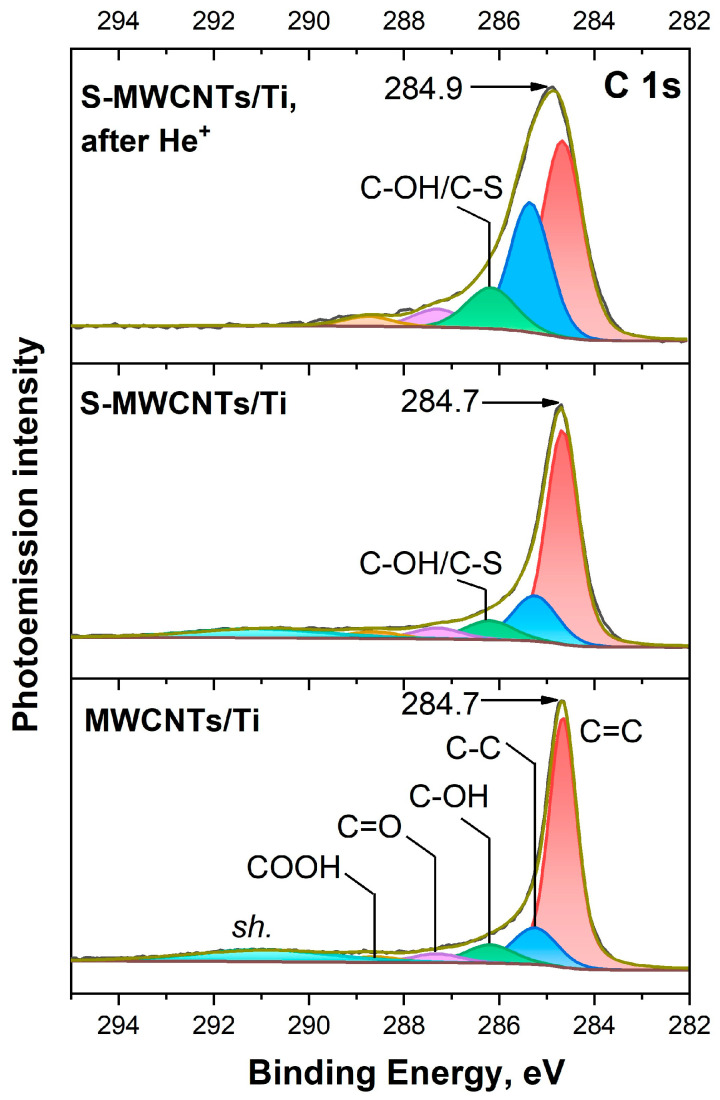
C 1*s* PE spectra of MWCNTs/Ti as well as S-MWCNTs/Ti before and after irradiation by He^+^ ions.

Figure 4 displays the C 1*s* PE spectra of MWCNTs/Ti and S-MWCNTs/Ti before and after ion beam treatment. The PE spectrum of the MWCNTs/Ti consists of five PE lines that correspond to different types of carbon: graphite-like sp^2^ C=C (~284.7 eV), diamond-like sp^3^ C–C and/or carbon located near OCFGs [C*–C(O)] (~285.3 eV), carbon in C–OH groups (~286.2 eV), C=O (~287.3 eV) groups, and COOH– groups (~289.0 eV) [14,26,41,42]. In addition, there is another broad PE band, designated as *sh* (~291.0 eV). It signifies a satellite observed in the C 1*s* spectra of sp^2^-carbon atoms in highly graphitized systems associated with the π→π* shake-up process simultaneously with C 1*s* photoionization [26,41]. 

When moving to the S-MWCNTs/Ti sample, no significant spectral changes were observed except for a slight increase in the full width at half maximum (FWHM) of the C 1*s* PE spectrum from 0.7 to 0.9 eV. This broadening was mainly caused by the intensity growth of the C–C component. In other words, a slight increase in defectiveness was detected, which was apparently associated with the process of the S-MWCNTs preparation. At the same time, Figure 4 clearly demonstrates that as a result of irradiation of the S-MWCNTs/Ti system, there were significant changes in the shape of the C 1*s* PE spectrum. Specifically, an increase in the FWHM of the spectrum from 0.9 to 1.5 eV can be observed, which was caused by a redistribution of the relative intensities of its components. Additionally, there is no evidence of a shake-up satellite at 291.0 eV. These findings suggest variation in the quantity of carbon atoms in various chemical states in the system, the possible creation of chemical bonds between MWCNTs’ carbon atoms and other system atoms, and considerable defect formation in the walls of nanotubes. Detailed analysis of the spectra showed that alterations in the FWHM of the C 1s spectrum in the irradiated S-MWCNTs/Ti sample were due to redistribution of the relative intensities of various bands, such as C=C (sp^2^-carbon) and C–C bands and C–OH, C=O and COOH– bands corresponding to carbon atoms bonded to oxygen atoms (Figure 4). At the same time, it can clearly be seen that there was a significant increase in the intensity of the irradiated samples at binding energies around 286.1–286.6 eV compared to MWCNTs/Ti and S-MWCNTs/Ti. This increase cannot be explained only by the formation of additional C–OH bonds. According to the literature [43,44,45], in this energy region, there are not only C–OH states, but also C–S states, which correspond to chemical bonds between carbon and sulfur atoms. However, due to the close proximity of the energy positions of the C–OH and C–S PE signals, it is not possible to correctly separate them. Therefore, in our case, they are combined into a single C–OH/C–S component.

Thus, changes in the C 1*s* PE spectrum of the irradiated S-MWCNTs/Ti system indicate that the outer walls of nanotubes undergo significant structural damage as a result of ion irradiation. This leads to an increase in the number of wall defects and the attachment of OCFGs at these defect sites. In addition, the formation of direct C–S chemical bonds between the defective carbon walls of MWCNTs and sulfur atoms has been noted.

**Figure 5:**
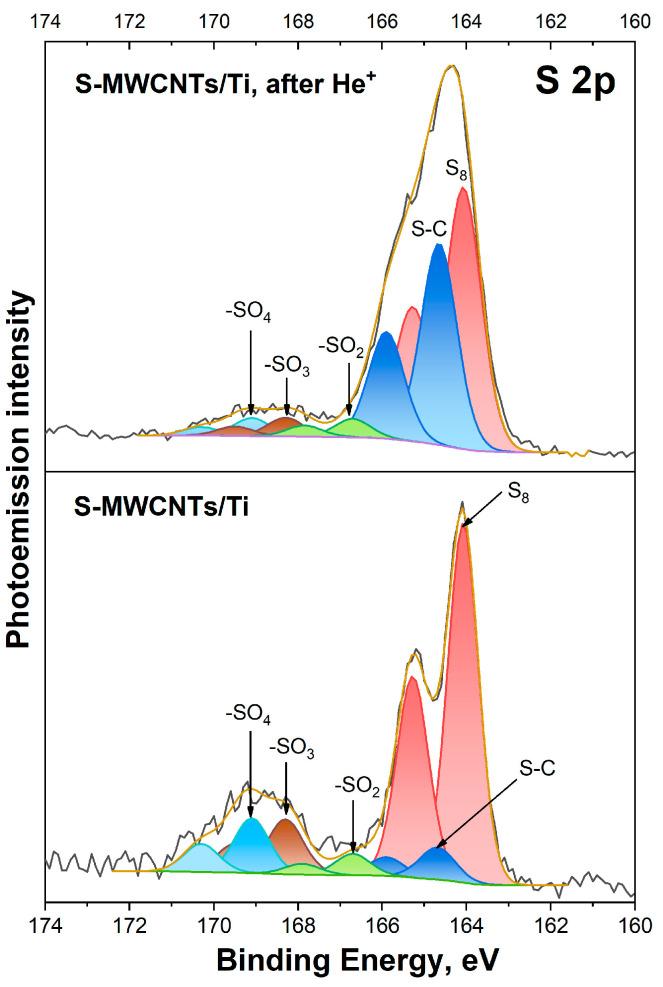
S 2*p* PE spectra of S-MWCNTs/Ti before and after irradiation by He^+^ ions.

Now, let us move on to examining the S 2*p* spectra of S-MWCNTs/Ti samples before and after irradiation with He^+^ ions, as shown in Figure 5. In the S 2*p* PE spectrum of the initial S-MWCNTs/Ti system, five spin-doublet (S 2*p*_3/2_, S 2*p*_1/2_) PE bands can be distinguished. These bands are marked with different colors and correspond to different chemical states of sulfur atoms: the elemental form S_8_ (~164.1 and 165.3 eV) and S–C bonds with carbon (~164.7 and 165.9 eV), as well as S–O bonds with oxygen in -SO_2_ (~166.7 and 167.9 eV), -SO_3_ (~168.3 and 169.5 eV) and -SO_4_ (~169.1 and 170.3 eV) groups [13,45,46,47,48,49]. The results of the analysis of the S 2*p* PE spectra using these bands for the initial and irradiated S-MWCNTs/Ti system are presented in Table 2. From these data, we can see that in the initial system, most of the sulfur is present in the elemental form (~65%), accounting for approximately 1.11 at.%. The rest of the sulfur is found in chemical bonds with oxygen (~27%), representing 0.46 at.%, and carbon (~6%), accounting for 0.11 at.%. Irradiation of S-MWCNTs on a titanium substrate with He^+^ ions results in a change in the shape and broadening of the S 2*p* PE spectrum. Both effects can be attributed to an increase in the intensity of the S–C PE band, which corresponds to the chemical bonds between sulfur and carbon atoms in the S-MWCNT walls. It should be noted that the relative intensity of this PE band compared to that in the spectrum of the initial S-MWCNTs/Ti system increases by 5.7 times. In terms of the total sulfur concentration, this corresponds to 4.2 at.%. This result can be explained by two factors: (i) the formation of point structural defects, mainly mono- and divacancies, under the action of an ion beam, which leads to an increase in the free energy of the surface of MWCNTs, and (ii) the redeposition of sulfur under the same conditions, with the subsequent formation of direct S–C chemical bonds near these defects. Also, as a result of irradiation, the formation of thiophene-like defects in the structure of the walls of MWCNTs cannot be excluded [50,51,52]. However, if they are formed, their number will be insignificant, and they will not affect the electrochemical characteristics of S-MWCNTs.

**Figure 6:**
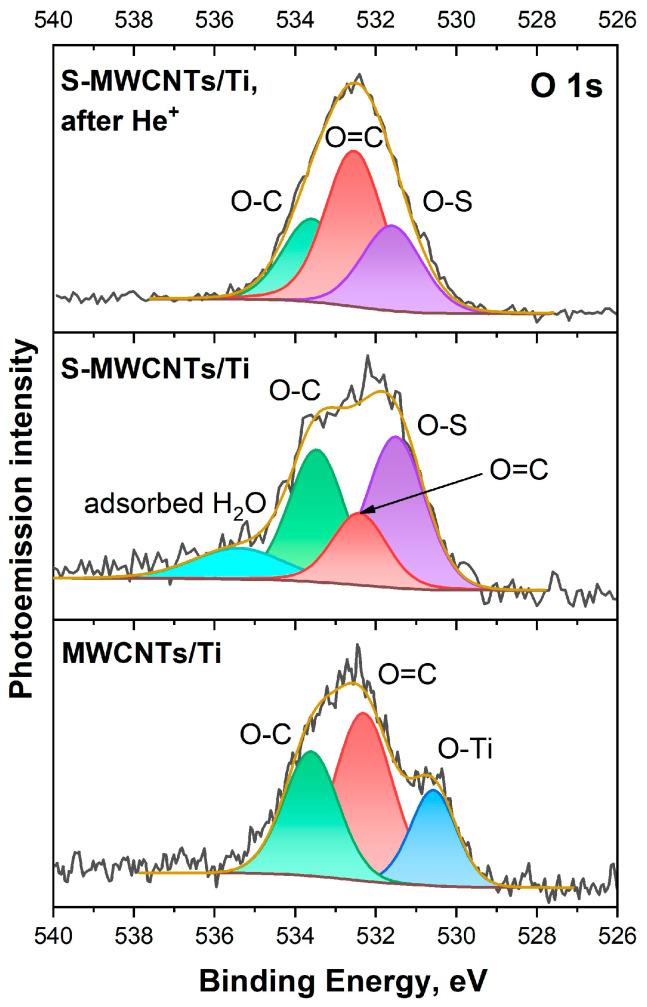
O 1*s* PE spectra of MWCNTs/Ti as well as S-MWCNTs/Ti before and after irradiation by He^+^ ions.

Finally, let us move on to examining the O 1*s* PE spectra of the studied systems, as presented in Figure 6. In the O 1*s* PE spectrum of the MWCNTs/Ti system, three distinct components can be identified, which correspond to oxygen atoms within TiO_2_ (~530.6 eV), as well as in oxygen-containing carbon groups with either double or single chemical bonds between carbon and oxygen atoms: C=O (~532.3 eV) and C−O (~533.5 eV) [26,53,54]. The presence of the Ti−O band in the O 1*s* PE spectrum may be due to the presence of scratches on the probing area, where elements of the titanium substrate without an MWCNTs layer are present. When moving to the initial S-MWCNTs/Ti sample, there is an increase in the FWHM value of the PE spectrum from 2.49 to 3.14 eV, which is associated with a change in the intensities of the C=O and C−O bands, as well as the appearance of two new bands at 531.6 and 535.4 eV. According to studies [44,55,56,57,58], the PE band at 531.6 eV is associated with various sulfur oxides (designated as S−O). The high-energy band at 535.4 eV, on the other hand, is likely due to water vapor adsorbed on the surface of the S-MWCNTs/Ti sample after its preparation.

Irradiation of the S-MWCNTs/Ti system with He^+^ ions results in a change in the shape of the O 1*s* PE spectrum, as well as a decrease in the FWHM value from 3.14 to 2.67 eV (Figure 6). This result can be attributed to a significant increase in the intensity of the C=O band, which corresponds to the presence of oxygen in carbon compounds with a double chemical bond. Furthermore, after irradiation, the S–O PE band associated with sulfur oxides on the surface of S-MWCNTs is retained, while the high-energy component corresponding to adsorbed water, on the contrary, disappears. Analyzing the data obtained, it can be concluded that as a result of irradiation with He^+^ ions, a large number of C=O groups are formed on the surface of MWCNTs, and water is desorbed from their outer walls into the implantation chamber. It is also important to note that, although the intensity of the S−O band decreases when compared to the initial S-MWCNTs/Ti system, a significant increase in the number of S-O bonds can be observed when the data are recalculated relative to the total oxygen content. Specifically, the content of these bonds increases from 1.3 to 3.4 at.%, which is a 2.6-fold increase. In other words, this may indicate ion-stimulated formation of additional S−O groups on the defective surface of MWCNTs.

### 3.4. Electrocatalytic Studies

In this section of the study, a comparative analysis of the catalytic activity of the initial and irradiated S-MWCNTs/Ti systems in the ORR and the oxidation reaction of hydrogen peroxide (H_2_O_2_) at different p*H* values was performed to determine their potential use as materials for an electrochemical oxygen and hydrogen peroxide sensor.

**Figure 7:**
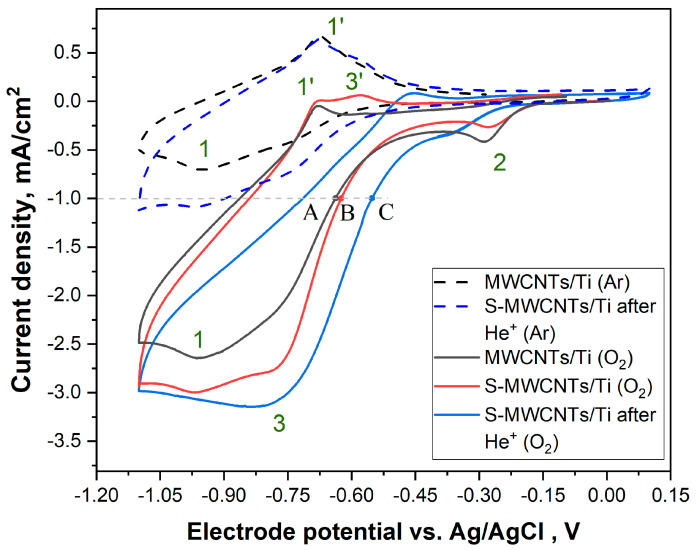
Cyclic voltammograms for the MWCNTs/Ti. The initial and irradiated S-MWCNTs/Ti systems recorded in a 0.1 M KOH electrolyte at a scan rate of 10 mV/s (the horizontal dotted line indicates the onset current). The atmospheres in which the measurements were performed are given in brackets: argon (Ar) and oxygen (O_2_).

Figure 7 shows the cyclic voltammograms (CVs) that were recorded for the MWCNTs/Ti, the initial and irradiated S-MWCNTs/Ti systems in a 0.1 M KOH electrolyte saturated with either argon or oxygen. The current densities presented here were calculated based on the geometric area of the electrode, which was 0.42 cm^2^. All samples were studied under similar conditions to allow comparison. 

From Figure 7 (dashed curve), it is evident that when the solution was saturated with argon, a cathodic peak, *1*, was observable on the CV curve of the MWCNTs/Ti material (black dotted curve) at –0.94 V, followed by anodic peak *1’* at –0.68 V, which can likely be attributed to the surface functional groups of the multi-walled carbon nanotubes. The CV curve of the He⁺-irradiated S-MWCNTs/Ti material (blue dashed curve) shows the same peaks as that of MWCNTs/Ti, indicating that no additional redox groups are introduced. However, for the irradiated system, the cathodic peak *1* current rises slightly due to the increase in the internal surface area or the electrocatalytic reduction of trace oxygen, which is a result of the defects introduced by the ion irradiation process. These surface groups, such as carboxyl or hydroxyl groups, may undergo redox transformation, which is detected as the corresponding peaks in the voltammogram. 

Upon saturation of the solution with oxygen, cathodic peaks *2* and *3* appears on all CV curves (most clearly for He^+^-irradiated S-MWCNTs/Ti (O_2_) (the blue curve)) at an electrode potential of –0.8 V vs. Ag/AgCl. These peaks indicate ORR activity, peak *2* showing the adsorption-type pre-peak, which indicates the reduction of adsorbed oxide species, and peak *3*, corresponding to diffusion-controlled reduction of dissolved oxygen, which is the target process in ORR-based systems. All samples tested, including the MWCNTs/Ti system, display some degree of catalytic activity for the ORR [58]. Interestingly, when comparing the MWCNTs/Ti (black curve) to the S-MWCNTs/Ti system (red curve), there are no substantial differences in the ORR activity in terms of the onset potential of oxygen electroreduction. In other words, sulfur does not contribute notably to the improvement of ORR performance in this sample. At the same time, an increase in current density up to 20% upon transition to the S-MWCNTs/Ti sample (this can be seen from the comparison of the peak currents of the *3* peaks) can be associated with an increase in the electrochemically active surface area, which is due to a change in morphology during the preparation of this system (see Figure 1d,e). However, in the case of the He⁺ ion-irradiated S-MWCNTs/Ti system, significant changes can be observed. Specifically, the onset potentials of the ORR measured as the potentials at the cathodic current –1 mA cm^−2^ are –0.65, –0.64 and –0.55 V for MWCNTs/Ti, S-MWCNTs/Ti and He⁺-irradiated S-MWCNTs/Ti, respectively. In other words, a positive shift of ~0.1 V of the onset potential for the He⁺-irradiated material in comparison to the MWCNTs/Ti and S-MWCNTs/Ti samples (points *A*, *B* and *C* in Figure 7 (the horizontal dotted line indicates the current chosen to pick the onset potential)). This shift suggests a notable improvement in the catalytic efficiency of the ORR in the irradiated system. The shift in the onset potential is likely due to changes in the interactions between sulfur and carbon atoms on the nanotube surfaces caused by ion irradiation. 

Based on the XPS data (Figure 5, Table 1 and Table 2), it was confirmed that sulfur atoms form direct S−C bonds with carbon atoms at the sites of defects on the MWCNT surfaces as a result of the irradiation process. This bonding supports the hypothesis that the changes observed in the voltammetric behavior, particularly the shift in the onset potential, are due to the formation of these direct S−C bonds. The presence of these bonds suggests that sulfur atoms are not merely passively attached to the MWCNT surfaces but actively participate in the catalytic process. Thus, sulfur atoms likely act as active catalytic sites for the ORR, enhancing the electrochemical activity of the helium-irradiated S-MWCNTs/Ti system.

In addition to ORR catalytic activity, the distinctive structural features of the He⁺-irradiated S-MWCNTs/Ti system make it an ideal candidate as a catalyst for the H₂O₂ oxidation reaction. The introduction of sulfur atoms and their interaction with the carbon nanotube surface due to (after) ion irradiation create a unique electronic environment that potentially promotes more effective interaction with H₂O₂ molecules. 

**Figure 8:**
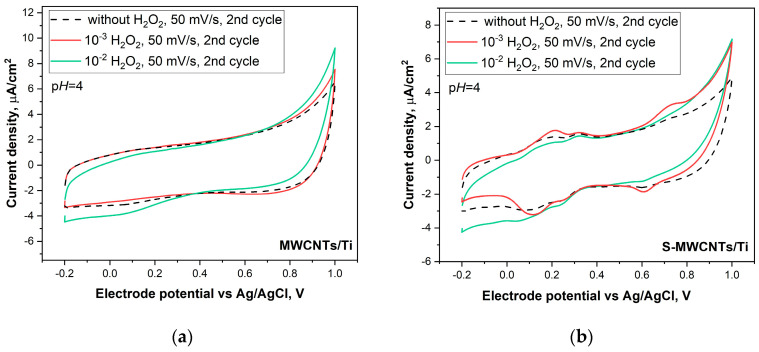
Cyclic voltammograms of MWCNTs/Ti (**a**) and S-MWCNTs/Ti (**b**) at a scan rate of 50 mV/s in buffer solutions with a p*H* of 4 with different (10^−2^ and 10^−3^ M) H_2_O_2_ concentrations.

**Figure 9:**
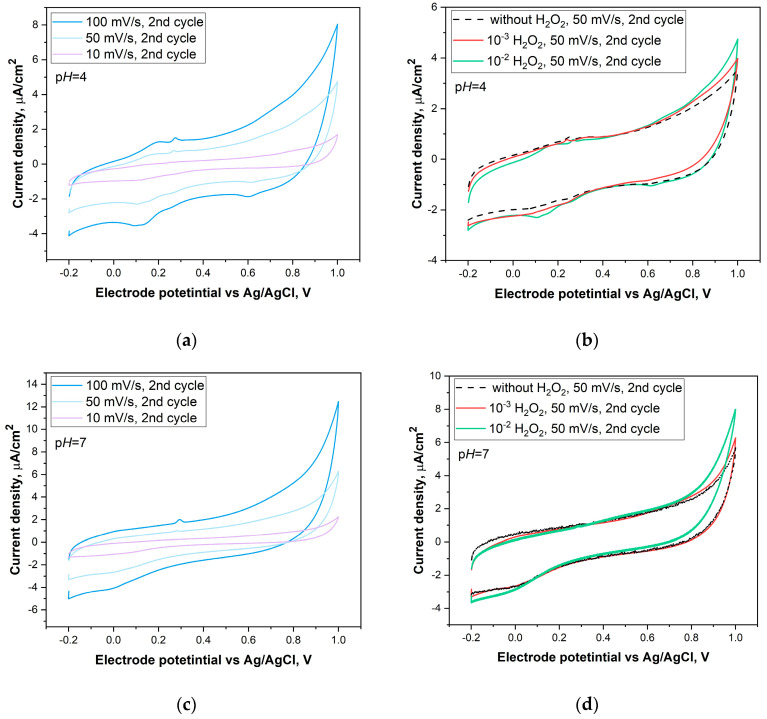

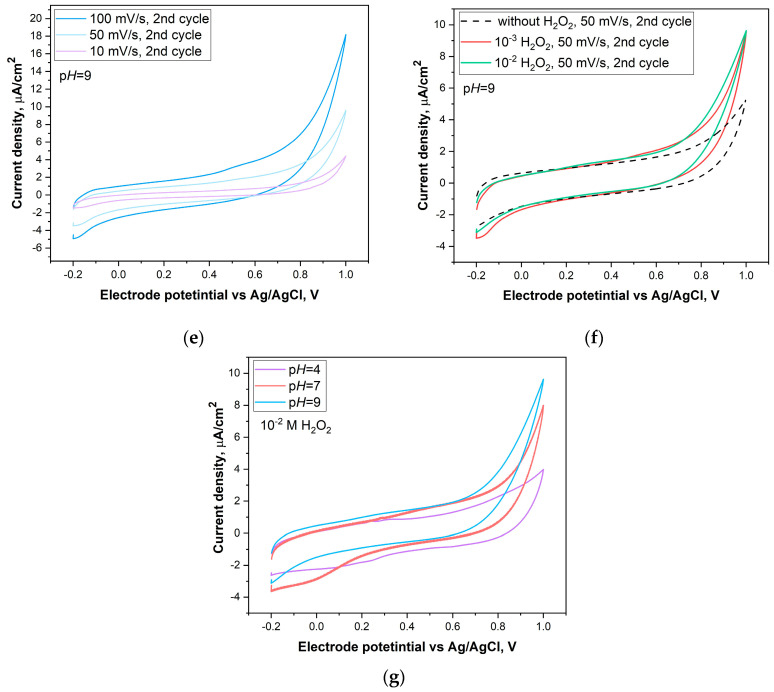
Cyclic voltammograms of helium-irradiated S-MWCNTs/Ti electrodes at different scan rates (10, 50 and 100 mV/s) in buffer solutions with p*H*s of 4, 7 and 9 without H_2_O_2_ (**a**,**c**,**e**) and at a scan rate of 50 mV/s in buffer solutions with different (10^−2^ and 10^−3^ M) H_2_O_2_ concentrations (**b**,**d**,**f**), as well as a comparison of CV curves in buffer solutions with p*H*s 4, 7 and 9 with a concentration of 10^−2^ M H_2_O_2_ (**g**).

To evaluate the potential of this system for H₂O₂ oxidation, CV experiments were performed in phosphate-buffered solutions at p*H* 4, 7 and 9 in both the presence and absence of H₂O₂. In the studied potential range, H_2_O_2_ oxidation proceeds in a kinetic regime; thus, to study the electrocatalytic properties of the synthesized materials, we should use the Electrochemically Available Surface Area (ECSA) to compare data between different systems. ECSA was determined by cyclic voltammetry (CV) using the double-layer charging currents recorded at a potential of 0.4 V. At the specified potential of 0.4 V, the measured current predominantly reflects the double-layer charging, allowing for the estimation of the double-layer capacitance (*C_dl_*) (see ESI for non-normalized data (Figures S1 and S2). This capacitance was used for calculating the ECSA, using the following relationship:

*ECSA = C_dl_/C_specific_*

where *C_specific_* represents the specific capacitance of the carbon material, typically ranging from 20 to 30 µF/cm^2^ for carbon-based electrodes. We used 20 µF/cm^2^ for all studied materials. The calculated ECSA values were used to normalize the current providing current densities (plotted in Figure 8 and Figure 9).

The CV of the MWCNT/Ti material (Figure 8a, black dashed curve) shows only a capacitive response in the solution without peroxide, and the addition of H_2_O_2_ has no effect up to a concentration of 10^−2^ M. After the addition of 10^−2^ M H₂O₂, the anodic current slightly increased. When going to the S-MWCNTs/Ti sample (Figure 8b), the CV curves show the presence of reversible peaks in the vicinity of 0.2 V, which are located outside the region of peroxide oxidation. Moreover, adding 10^-3^ M H₂O₂ to the solution leads to an increase in the anodic current at potentials above 0.6 V for MWCNTs/Ti and S-MWCNTs/Ti, but switching to 10^−2^ M H₂O₂ does not produce a further noticeable increase in current (Figure 8b), indicating limited availability of catalytically active areas in the material not irradiated by the He⁺ beam. The CV curves for He⁺-irradiated S-MWCNTs/Ti, recorded at different scan rates in a p*H* 4 buffer (Figure 9a), show a capacitive current at all scan rates, indicating stable charge storage behavior. In the absence of H₂O₂, small redox peaks can be observed near 0.2 V, which, as in the case of the intact S-MWCNTs/Ti material, are attributable to the redox activity of OCFGs on the MWCNT surfaces, introduced during electrode preparation and further modified by helium ion irradiation. The rise in current with increasing scan rates (from 10 to 100 mV/s) highlights efficient charge transport, a key feature of MWCNTs, which appeared not to be altered by the defective and sulfur-functionalized surface of the material.

Upon the addition of H₂O₂, the CV curves (Figure 9b) revealed a distinct rise in anodic oxidation currents between 0.6 and 1.0 V, demonstrating that the material was active in the electrooxidation of H₂O₂ in the p*H* 4 buffer. Since water oxidation with oxygen evolution occurs within the same potential range, it is logical to compare the increase in current relative to the background when no hydrogen peroxide is present. The increase in current compared to the background at p*H* 4 was relatively modest, however; unlike the non-irradiated sample, it did grow as the H₂O₂ concentration increased. At p*H* 7 (Figure 9c,d), the current increase was significantly higher, indicating enhanced activity under these conditions. At p*H* 9, while the anodic current initially rose sharply and reached higher values compared to p*H* 7 for the diluted peroxide solution, a saturation effect with increasing H₂O₂ concentration became apparent (Figure 9g). At an H₂O₂ concentration of 10^−2^ mol/L, the maximum anodic current in the vicinity of 1 V nearly matched that observed at 10^−3^ mol/L (Figure 9e,f). These observations suggest that although the electro-oxidation current of H₂O₂ increases with the p*H* of the buffer solution, the activity of the He⁺-irradiated S-MWCNTs/Ti system plateaus under strongly alkaline conditions. This plateau indicates that the active sites on He⁺-irradiated S-MWCNTs/Ti may become fully utilized, limiting further increases in current despite the addition of more H₂O₂.

In summary, the electrochemical investigation of the He⁺-irradiated sulfur-functionalized multi-walled carbon nanotubes revealed significant catalytic activity for both the ORR and hydrogen peroxide (H_2_O_2_) oxidation. The current value increased systematically with increasing H₂O₂ concentration and p*H*, with the highest activity observed at p*H* 9. These results indicate that the structural modifications, including sulfur incorporation and defect formation, significantly enhance the material’s catalytic efficiency for H₂O₂ oxidation, making it a promising candidate for electrocatalytic applications in both ORR and H₂O₂ detection. Therefore, the S−C bonds at these defective sites likely contribute to a more localized and effective interaction with H₂O₂, which, being one of the products of the ORR in carbon materials, requires a similar set of adsorption centers. As a result, these defects can serve as active sites that enhance electron transfer to peroxide molecules, thus increasing the catalytic efficiency for reactions such as H₂O₂ oxidation.

## 4. Conclusions

Based on the conducted studies using a combination of complementary analytical methods, such as SEM, Raman, EDX and XPS, a detailed characterization of the structure of sulfur-containing multi-walled carbon nanotubes (S-MWCNTs) on a titanium substrate was performed before and after helium ion treatment. It was established that exposure to high-energy helium ions leads to an increase in defects within the nanotube walls and the attachment of oxygen-containing functional groups, including SO_x_ groups, near these defects. Additionally, the sulfur concentration on the nanotube surface significantly increased (by approximately 6.4 times) compared to the untreated system, which is attributable to the sputtering and redeposition of sulfur across the surface of individual nanotubes under the influence of the ion beam. One of the key findings was the discovery of ion-stimulated formation of direct S–C chemical bonds, likely involving carbon atoms in the defected areas of the nanotube walls. The concentration of these S–C bonds increased 38-fold compared to the untreated system, reaching 4.2 at.%.

In terms of electrochemical performance, cyclic voltammetry experiments confirmed the potential of the material as an efficient electrocatalyst. After irradiation, the S-MWCNTs/Ti exhibited a positive shift in ORR onset potential by 0.1 V compared to MWCNTs/Ti and S-MWCNTs/Ti, indicating enhanced catalytic activity due to He^+^ irradiation after sulfur incorporation. Furthermore, the irradiated S-MWCNTs also showed a significant increase in anodic current during hydrogen peroxide oxidation, especially at higher p*H* values, confirming their potential application as efficient sensors for H_2_O_2_ in different buffer solutions.

These results demonstrate the significant influence of helium ion irradiation on the structural and electrochemical properties of the S-MWCNTs/Ti system. The obtained material has great potential for use in electrochemical sensors, particularly for the detection of oxygen and hydrogen peroxide. Further optimization of these materials may pave the way for their use in various catalytic and sensor applications.

## Figures and Tables

**Table 1:** Chemical composition of samples according to EDX and XPS data.

Sample	Concentration, at. %			
	[C]	[O]	[S]	[Ti]
**EDX**				
MWCNTs/Ti	80.75	5.41	-	13.84
S-MWCNTs/Ti before irradiation	80.34	5.71	0.56	13.39
S-MWCNTs/Ti after irradiation	80.32	6.50	0.68	12.50
**XPS**				
MWCNTs/Ti	93.80	5.21	-	0.99
S-MWCNTs/Ti before irradiation	94.92	3.40	1.68	-
S-MWCNTs/Ti after irradiation	76.10	13.11	10.79	-

**Table 2:** The results of a five-component fitting of the spectral distribution in the S 2*p* PE spectra and an evaluation of the concentrations of various sulfur-containing components in S-MWCNTs/Ti before and after He^+^ ion irradiation.

Sample	S_8_	S–C	-SO_2_	-SO_3_	-SO_4_
**Relative Component Intensity, %**					
S-MWCNTs/Ti before irradiation	65.6	6.8	4.4	11.7	11.5
S-MWCNTs/Ti after irradiation	50.4	38.9	3.6	3.6	3.5
**Concentration, at.%**					
S-MWCNTs/Ti before irradiation	1.11	0.11	0.07	0.20	0.19
S-MWCNTs/Ti after irradiation	5.43	4.20	0.39	0.39	0.38

## Data Availability

Data are contained within the article and Supplementary Materials.

## Supplementary Materials

The following supporting information can be downloaded at: https://www.mdpi.com/article/10.3390/nano14231948/s1, Figure S1: Cyclic voltammograms of MWCNTs/Ti (a) and S-MWCNTs/Ti (b) at a scan rate of 50 mV/s in buffer solutions with p*H* of 4 with different (10^−2^ and 10^−3^ M) H_2_O_2_ concentrations (raw data); Figure S2: Cyclic voltammograms of helium-irradiated S-MWCNTs/Ti electrode at different scan rates (10, 50 and 100 mV/s) in buffer solutions with pH of 4, 7 and 9 without H_2_O_2_ (a,c,e) and at a scan rate of 50 mV/s in buffer solutions with different (10^−2^ and 10^−3^ M) H_2_O_2_concentrations (b,d,f) (raw data).
