# Supplementary material for: Structure and Electrocatalytic Properties of Sulfur-Containing Multi-Walled Carbon Nanotubes on a Titanium Substrate Modified by a Helium Ion Beam
Source: Nanomaterials (Basel). 2024 Dec 4;14(23):1948. doi: 10.3390/nano14231948 (PMC11642979; doi:10.3390/nano14231948)
Supplement: Supplementary file 1 [file nanomaterials-14-01948-s001.zip › nanomaterials-3290344-supplementary.pdf]

nanomaterials

Supplementary information

Article

Structure and electrocatalytic properties of sulfur-containing multi-walled carbon nanotubes on a titanium substrate modified by helium ion beam

Petr M. Korusenko [1,2,*], Egor V. Knyazev [2,3], Alexander S. Vinogradov [4], Ksenia A. Kharisova [1], Sofya S. Filippova [1], Ulyana M. Rodionova [1], Oleg V. Levin [1], Elena V. Alekseeva [1]

1 Electrochemistry Department, St. Petersburg State University, 7/9 Universitetskaya nab., 199034 St. Petersburg, Russia
2 Department of Physics, Omsk State Technical University, 11 Mira prosp., 644050 Omsk, Russia
3 Laboratory of Physics of Nanomaterials for Chemical Current Sources, Omsk Scientific Centre Siberian Brunch of Russian Academy of Science, 15 Karl Marx prosp.,644013 Omsk, Russia
4 Department of Solid State Electronics, V.A. Fock Institute of Physics, St. Petersburg State University, 7/9 Universitetskaya nab., 199034 St. Petersburg, Russia
* Correspondence: korusenko_petr@mail.ru

Abbreviations and parameters

MWCNTs –multi-walled carbon nanotubes
S-MWCNTs – sulfur-containing multi-walled carbon nanotubes
S-MWCNTs/Ti – S-MWCNTs placed on a titanium substrate

Electrochemical raw data

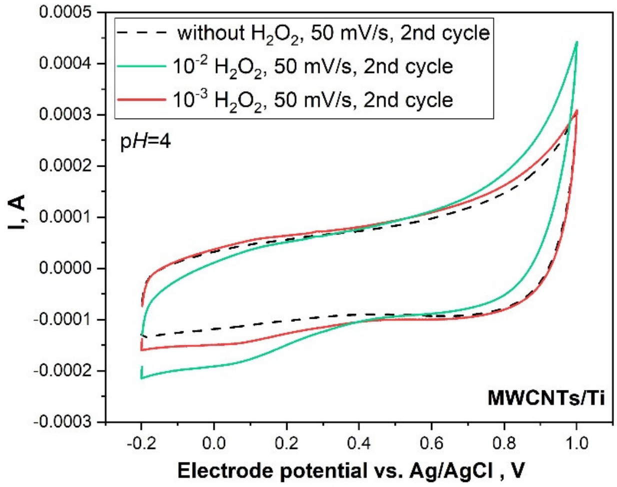

(a)

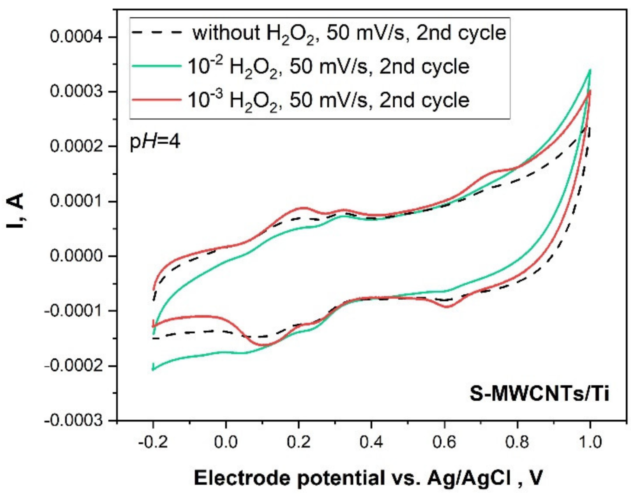

(b)

Figure S1. Cyclic voltammograms of MWCNTs/Ti (a) and S-MWCNTs/Ti (b) at a scan rate of 50 mV/s in buffer solutions with p*H* of 4 with different (10^{-2} and 10^{-3} M) H_2O_2 concentrations (raw data).

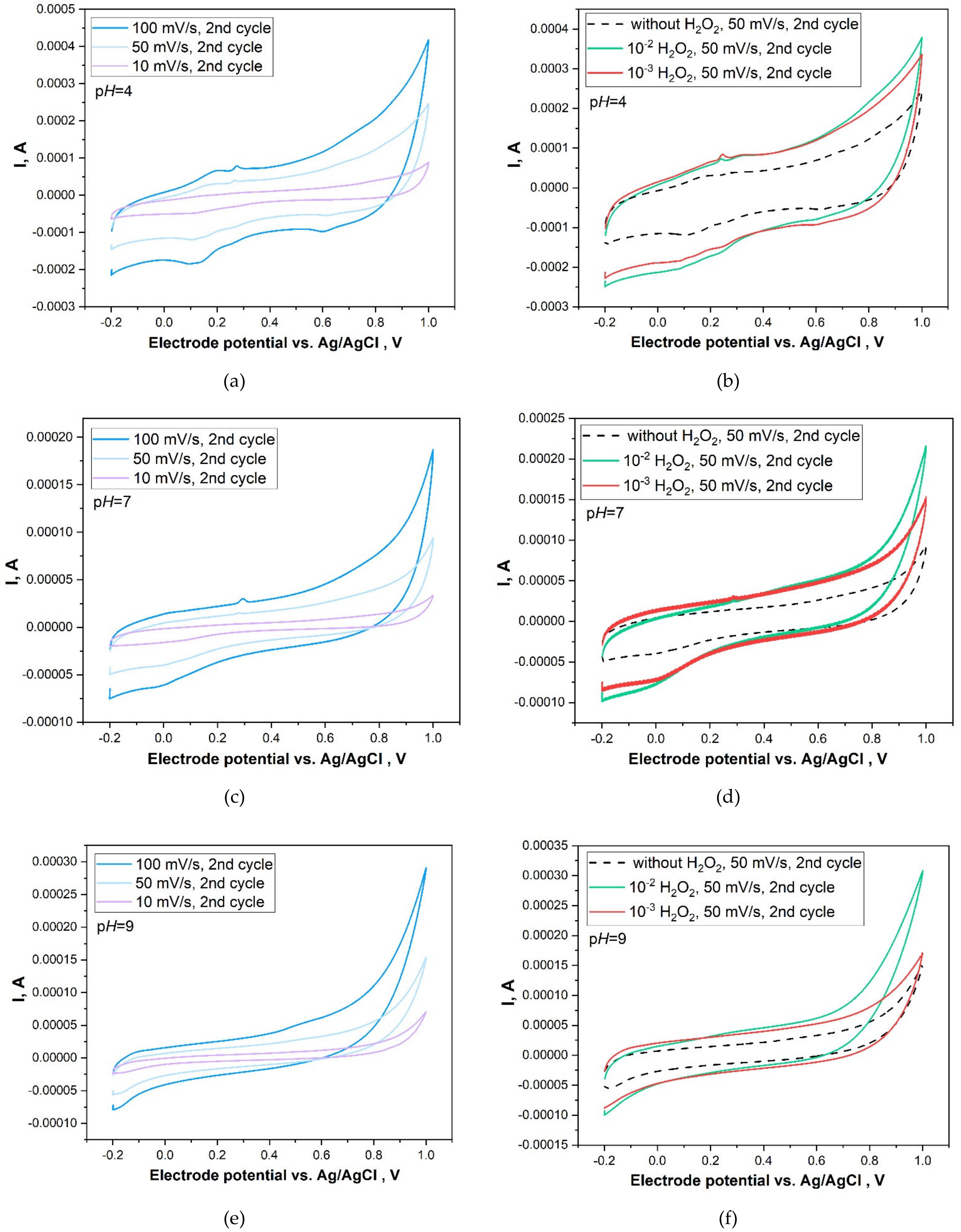

Figure S2. Cyclic voltammograms of helium-irradiated S-MWCNTs/Ti electrode at different scan rates (10, 50 and 100 mV/s) in buffer solutions with p*H* of 4, 7 and 9 without H_2O_2 (a), (c), (e) and at a scan rate of 50 mV/s in buffer solutions with different (10^{-2} and 10^{-3} M) H_2O_2 concentrations (b), (d), (f) (raw data).
